# Application of Edible Coating Based on Liquid Acid Whey Protein Concentrate with Indigenous *Lactobacillus helveticus* for Acid-Curd Cheese Quality Improvement

**DOI:** 10.3390/foods11213353

**Published:** 2022-10-25

**Authors:** Agne Vasiliauskaite, Justina Mileriene, Epp Songisepp, Ida Rud, Sandra Muizniece-Brasava, Inga Ciprovica, Lars Axelsson, Liis Lutter, Elvidas Aleksandrovas, Ene Tammsaar, Joana Salomskiene, Loreta Serniene, Mindaugas Malakauskas

**Affiliations:** 1 Department of Food Safety and Quality, Veterinary Academy, Lithuanian University of Health Sciences, Tilzes Str. 18, LT-47181 Kaunas, Lithuania; 2 BioCC OÜ, Riia 181A-233, 50411 Tartu, Estonia; 3 Nofima-Norwegian Institute of Food, Fisheries and Aquaculture Research, P.O. Box 210, NO-1431 Ås, Norway; 4 Faculty of Food Technology, Latvia University of Life Sciences and Technologies, Rigas Str. 22A, LV-3002 Jelgava, Latvia; 5 Microbiology Research Laboratory, Food Institute, Kaunas University of Technology, K. Donelaicio Str. 73, LT-44249 Kaunas, Lithuania

**Keywords:** antimicrobial lactobacilli, edible coating, liquid acid whey protein concentrate, apple pectin, *Lactobacillus helveticus*, acid-curd cheese, quality improvement

## Abstract

Edible coatings as carriers for protective lactic acid bacteria (LAB) can enhance hygienic quality to dairy products. Thus, the aim of this study was to improve the quality of artisanal acid-curd cheese by applying liquid acid whey protein concentrate based edible coating with entrapped indigenous antimicrobial *Lactobacillus helveticus* MI-LH13. The edible fresh acid-curd cheese coating was composed of 100% (*w*/*w*) liquid acid whey protein concentrate (LAWPC), apple pectin, sunflower oil, and glycerol containing 6 log_10_ CFU/mL of strain biomass applied on cheese by dipping. The cheese samples were examined over 21 days of storage for changes of microbiological criteria (LAB, yeast and mould, coliform, enterobacteria, and lipolytic microorganism), physicochemical (pH, lactic acid, protein, fat, moisture content, and colour), rheological, and sensory properties. The coating significantly improved appearance and slowed down discolouration of cheese by preserving moisture during prolonged storage. The immobilisation of *L. helveticus* cells into the coating had no negative effect on their viability throughout 14 days of storage at 4 °C and 23 °C. The application of coating with immobilised cells on cheeses significantly decreased the counts of yeast up to 1 log_10_ CFU/g during 14 days (*p* < 0.05) of storage and suppressed growth of mould for 21 days resulting in improved flavour of curd cheese at the end of storage. These findings indicate that LAWPC-pectin formulation provided an excellent matrix to support *L. helveticus* cell viability. Acting as protective antimicrobial barrier in fresh cheeses, this bioactive coating can reduce microbial contamination after processing enabling the producers to extend the shelf life of this perishable product.

## 1. Introduction

Edible coatings are natural biodegradable layers on foods retaining the appearance, physicochemical properties, and freshness during their storage and serving as an improvement to conventional packaging [1]. Many protein-based edible coatings intended for cheese protection contain sweet whey proteins that in a majority of studies [2,3,4,5,6,7] are purchased in powdered form along with other coating ingredients using water as a solvent [8]. Acid whey, on the other hand, a main waste product of acid curd production, causes devastating environmental problems and, therefore, calls for reintroducing it back into production. Cheese factories, responding to the sustainability and environmental demands, are searching for the ways to repurpose acid whey, preferably in its liquid form [9]; therefore, ultrafiltration is most widely used by cheese factories to acquire liquid acid whey concentrate (LAWPC) from acid whey. Employing LAWPC with specific nutritional value and functional properties [10,11] as a basis for acid whey protein coating, and as a solvent for other coating ingredients, provides an opportunity to avoid subsequent energy-consuming spray drying process, which brings along environmental, health, and economic benefits [12,13,14]. In a liquid form, though it contains only 2.4–3.2% of whey protein—not ideal for coating production—thus, there is a need to supplement it with biopolymers (such as agro-industrial waste pectin) and lipids to make it work as a protectant layer on cheese surface.

Prolonging the shelf life of the product along with reducing food waste is another emerging need of modern society [15]. Fresh acid-curd or farmer’s cheese has long been a well-known artisanal dairy product, which, due to prevailing manual operations, has a short shelf life of 8–10 days [14]. Mould contamination of such cheese during storage is a problem for the small dairy farmers wrapping cheese to be sold in paper, which does not add up to the shelf life of this cheese [16]. It was reported that the shelf life of a product can be prolonged not only by reducing respiration with the help of main edible coating components [17] but also by addition of antimicrobial protectants to the coating formulations. Indigenous, probiotic-type lactic acid bacteria (LAB) are one of them, demonstrating protective properties [18,19,20] and antimicrobial potential [21]. Various immobilisation or encapsulation technologies have been widely studied underlining the impact of materials, environmental conditions, and individual strain properties on the survival of such LAB [22,23,24]. Preparation of bioactive coatings with protective LAB incorporated is an innovative field in the dairy industry, and therefore, only a small number of studies on this topic are available [25]. Coating formulations supporting LAB survival [26] could be a good aid in not only ensuring microbiological safety but also enhancing the flavour of such type of cheese.

To date, there is no research carried out on the application of LAWPC and pectin as a base in edible coating production and as a vehicle for protective LAB strains. Therefore, the aim of this study was to improve the quality of artisanal acid-curd cheese by applying liquid acid whey protein concentrate pectin-based edible coating with entrapped indigenous antimicrobial *Lactobacillus helveticus* MI-LH13. Simultaneously, the survival of *L. helveticus* MI-LH13 immobilised in the edible coating and the strain’s impact on the characteristics of this cheese were evaluated.

The production of edible coating by incorporating protective lactobacilli as natural antimicrobial agents in a matrix based on LAWPC could be an excellent opportunity to reuse whey in its liquid form in the factory that produced it. Applying produced bioprotective coating on fresh curd cheese by spraying or dipping could be an alternative or improvement to conventional cheese packaging, enhancing cheese sensory perception, preventing spoilage, and prolonging its shelf-life.

## 2. Materials and Methods

### 2.1. Materials

Liquid acid whey protein concentrate (LAWPC) was supplied from dairy plant AB Kauno pienas, Lithuania. LAWPC was produced by ultra-filtrating fresh acid bovine whey. The LAWPC (12.34% dry matter, presented 2.24% protein, 0.5% fat, and 5.55% other solids, pH 4.69) was frozen at −20 °C until use. Before coating preparation, the LAWPC was thawed at 4 °C.

Plasticiser glycerol (99% purity), thickener and emulsifier apple pectin, surfactant tween 80, and sunflower oil were supplied by Sigma-Aldrich, Darmstadt, Germany.

Indigenous *L. helveticus* MI-LH13 previously isolated from raw bovine milk was stored at −80 °C in MRS broth (Merck, Germany) in the presence of 30% glycerol. Antimicrobial properties of this strain and some of the technological properties relevant to the dairy industry were tested in vitro before the study [27].

The strain was revitalised in MRS broth (Biolife, Milano, Italy) by growing for 18 h at 30 °C until reaching 6 log_10_ CFU/mL. The biomass of the strain was harvested by centrifugation at 4000 rpm for 15 min at 4 °C.

Fresh acid-curd cheese (4.00% fat, 3.00% protein, 4.50% lactose, 8.10% non-fat solids, pH 6.60) determined with a milk analyser (Milko-Skan, FOSS ELEKTRIK, Hillerød, Denmark) was purchased from local artisanal dairy factory. It was made by a conventional method: standardised bovine milk was pasteurised, cooled down to 28 °C, and then commercial starter was added. After casein coagulation (12 h), the soured milk was heated up to 50 ± 1 °C (90 min) to form the curd. The curd was placed into cotton bags and pressed (approx. 30 min) until the required firmness of cheese was achieved. The resultant cheese samples (100 ± 5 g) were manually taken out of bags and stored in a refrigerator at 4 ± 1 °C for 24 h until coating application.

### 2.2. Coating and Film Preparation

Coating formulations were prepared according to the method described by Ramos et al. [5] with some modifications. In order to obtain the coating solution, 5% of glycerol (*w*/*w*), 2% of pectin (*w*/*w*), 0.2% of Tween (*w*/*w*), and 2% of sunflower oil (*w*/*w*) were added to the LAWPC, homogenised (15,000 rp/s, 3 min) for good dispersion, and solution was pasteurised in water bath (85 °C, 10 min). After cooling it down to 35 °C, *L. helveticus* biomass (0.2 g/100 g, respectively) was thoroughly mixed in.

Films were produced from the coating solution by pouring 5 mL of it in Petri dishes and letting them dry at 37 °C for 24 h.

To evaluate the survival of incorporated strain, the coating solution was kept at room temperature (23 ± 1 °C) and refrigerated (4 ± 1 °C) for 14 days. Films were kept in Petri dishes at 4 °C for 60 days.

### 2.3. Coating Application on Acid-Curd Cheese

Two coating formulations, plain coating (C) and coating with *L. helveticus* incorporated (C + Lh), were prepared and immediately applied by dipping fresh acid-cured cheese into the coating solution for a few seconds. Coated cheeses were placed on perforated metal trays to dry off for 60 min (12–14 °C) and then placed into paper bags and stored refrigerated at 4 ± 1 °C for 21 days in previously sanitised perforated plastic boxes.

### 2.4. Cheese Analyses

Cheese samples were analysed in triplicate on days 1, 7, 14, 18, and 21 for physicochemical, rheological, and microbiological changes. pH was directly measured with a pH meter (Sartorius Professional meter for pH Measurement, Goettingen, Germany). Titratable acidity, expressed as a percentage of lactic acid (g/100 g cheese), was determined according to the standard method ISO 11869:2012 [28].

Dry matter, moisture, acidity, fat, and protein determinations in samples were performed according to prescribed methods: dry matter and moisture ISO 5534:2004 [29], fat ISO 1735:2004 [30], lactose ISO 22662:2007 [31], and protein ISO 8968-3:2004 [32].

Colour characteristics (where L* = lightness, a* = red–yellow colour, and b* = blue–green colour) of cheese were assessed using a CIE L*a*b* system (1996) (Chromameter CR-400, Konica Minolta, Tokyo, Japan). A standard white plate was used to calibrate the equipment, with colour coordinates L_standard_ = 97.6, a_standard_ = 0.01, and b_standard_ = 1.60. The total colour difference (ΔE) was calculated as follows:

ΔE = [(L − L_0_)^2^ + (a − a_0_)^2^ + (b − b_0_)^2^]^1/2^,

where L_0_, a_0_, and b_0_ were values of day 1; and L, a, and b were the values measured throughout the storage period. Three readings were taken for each triplicate.

Textural properties of cheese samples were evaluated with the texture analyser CT3 (Brookfield, Middleboro, MA, USA) with a TA4/1000 cylinder (diameter of 38.1 mm D, 20 mm L, a stroke speed of 1 mm/s, and a strike depth of 10 mm).

For microbiological analysis, viable counts of microorganisms commonly found in this type of cheese were determined in triplicate on the selective media for each species at days 1, 7, 14, 18, and 21 of cheese storage. Microorganisms were enumerated according to the prescribed methods: total mesophilic LAB count ISO 15214:1998 [33], enterobacteria count ISO 21528-2:2017 [34], coliform count ISO 4832:2006 [35], and yeast and mould count ISO 6611:2004 [36]. Lipolytic bacteria were enumerated on MRS agar overlaid with lard as described by Tuynenburg Muys and Willemse (1965) [37].

Sensory analysis was conducted on days 1, 7, 14, and 18 of cheese storage upon previous confirmation of microbiological safety. Sensory evaluation was carried out in the sensory room by a trained panel of 7 members (both sexes, ages ranging between 20 and 50 years old). The panel had previously been selected and trained by guidelines of ISO 8586:2012 [38]. Prior to assessment, samples were coded with 3-digit randomised numbers and served at room temperature. Cheese samples (5 × 2 × 2 cm blocks) were randomly presented to the panel members in identical plastic plates. The scorecard for the sensory evaluation of curd cheese was designed according to Bodyfelt et al. (1988) [39] as follows: 50 points for flavour, 40 points for body and texture, and 10 points for the appearance with the overall perception of 100 points. Cheeses were considered to be acceptable if at least an overall score of 65 points was obtained.

### 2.5. Statistical Analysis

All data processing and analysis were performed by SPSS statistical package (Chicago, IL, USA, SPSS Inc., SPSS 24). The data were analysed using descriptive statistics (Explore) and two-way analysis of variance (ANOVA). Differences between pairs of means were assessed on the basis of confidence intervals using the Tukey test. All the statistical analysis was performed at 95% level of significance.

## 3. Results and Discussion

### 3.1. Survival of L. helveticus in the Coating and Film

**Figure 1:**
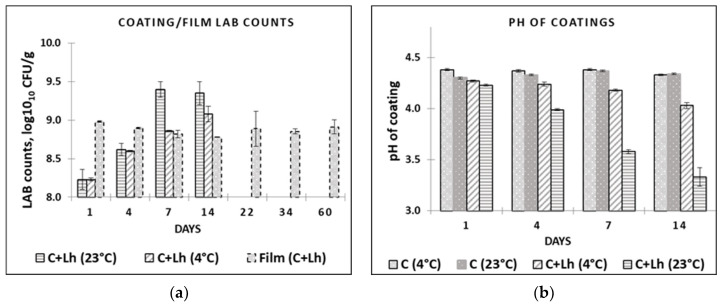
LAB counts (**a**) in coating solutions and films and pH; (**b**) in coating solutions at different temperatures (at 4 °C and 23 °C) during their storage. C—plain coating; C + Lh—coating solution with *L. helveticus*.

The impact of temperature on the survival of *L. helveticus* during storage in the coating solution (C + Lh) and the pH changes are shown in Figure 1a,b, respectively. *L. helveticus* grew to higher CFU at 23 °C (log_10_ 9.4) compared with 4 °C (log_10_ 9.0) during the 14 days, breaking down glucose that corresponded to faster decline in pH during storage at 23 °C, resulting in pH of 3.6 compared with 4.0 at 4 °C (*p* ≤ 0.05). This is in agreement with Perreira et al. (2016) [40] reporting temperature and strain dependencies when evaluating the survival rates of *Bifidobacterium animalis* Bb-12^®^ and *Lactobacillus casei*-01 in whey protein isolate coating at different temperatures (4 °C, 23 °C). In our study, LAB immobilised in the film demonstrated viability loss of ca. 0.5 log cycle within 14 days of storage at 4 °C that was kept stable thereafter (8.88 ± 0.03 on day 1 and 8.32 ± 0.03 on day 60; (*p* ≤ 0.05; Figure 1a). Perreira et al. (2016) [40] reported viability loss (3 log cycles, reaching 10^6^ CFU/g film until 60 d) at both 23 °C and 4 °C, noting the most marked decrease at 23 °C for both strains, with *B. animalis* demonstrating less decrease in its cell numbers (10^8^ CFU/g film). The combination of *L. helveticus*, low temperature [40], glycerol, whey proteins, and pectin as protectants [41] in LAWPC-pectin-based film ensured the survival of protective strain that could be used for the quality maintenance of perishable products. Since the initial low pH of AWPC (4.69) did not interfere with the survival of the strain in the coating and film, it can be recommended as a basis for coating preparations reducing negative environmental impact of acid whey as well.

### 3.2. Cheese Storage Trial

#### 3.2.1. Physicochemical Profile

Data in Table 1 illustrate the chemical changes in the coated curd cheeses during prolonged storage at 4 °C (23 days). We observed no impact of storage and treatment factors’ interaction on dry matter (DM) and moisture parameters, and only individual factors significantly impacted these parameters. Storage day, treatment, and their interaction had an impact on all other cheese components (*p* ≤ 0.005).

**Figure 2:**
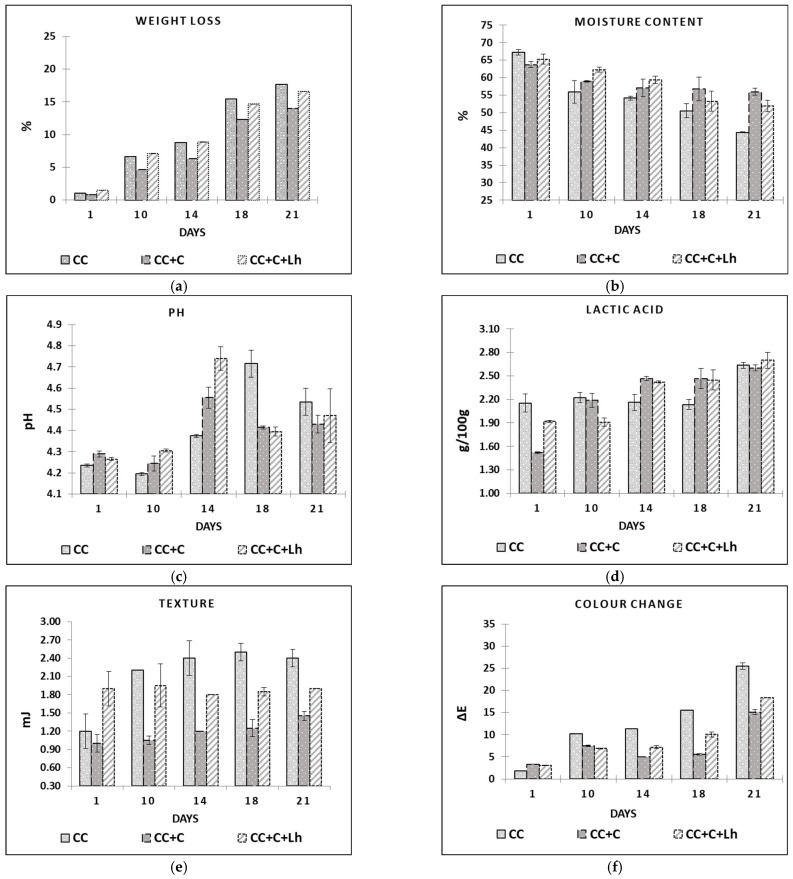
Weight loss (**a**), moisture content (**b**), pH (**c**), lactic acid content (**d**), texture (**e**), and colour change (**f**) in control acid-curd cheese (CC), coated acid-curd cheese (CC + C), coated acid-curd cheese with *L. helveticus* (CC + C + Lh) during 23 days of storage at 4–6 °C (mean values ± SD).

In the beginning of the experiment, both coated cheese treatments, with (CC + C + Lh) and without *L. helveticus* (CC + C), were significantly higher in fat and lactose content compared with control cheese (CC). Furthermore, the dry matter, protein, and fat contents of all cheese treatments significantly increased (*p* ≤ 0.0001) during the storage period mainly due to loss in moisture content (Figure 2b), while the use of coatings minimised moisture losses and hence improved the barrier properties (Figure 2b). The ability to preserve moisture during storage of the product is the most wanted parameter of edible coating [42]. Applied coating had no effect on initial moisture content—at day 1, no significant differences were noticed among samples. However, significant moisture loss was seen in uncoated control sample (CC, −19.7%, *p* < 0.05), whereas coated CC + C sample significantly lost less moisture (−13.9%, *p* < 0.05) during the 21 days of storage. This is in agreement with Mileriene et al. (2021) [43] stating that uncoated samples of acid-curd cheese significantly dried off faster than their coated counterparts. The addition of living strain to the coating, though, had a negative effect on its barrier properties. According to Ye et al. (2018) [44], the dispersion of bacterial cells in the film-forming solution destroys intermolecular interactions in the solution during film formation and increases the volume of voids in the living bacteria containing films, thereby slightly decreasing the tensile strength of the films but significantly increasing water vapour permeability.

The storage day, sample factors, and their interaction had a significant impact (*p* ≤ 0.0001) on instrumentally measured acidity, texture, and colour parameters (Figure 2a–f).

**Figure 3:**
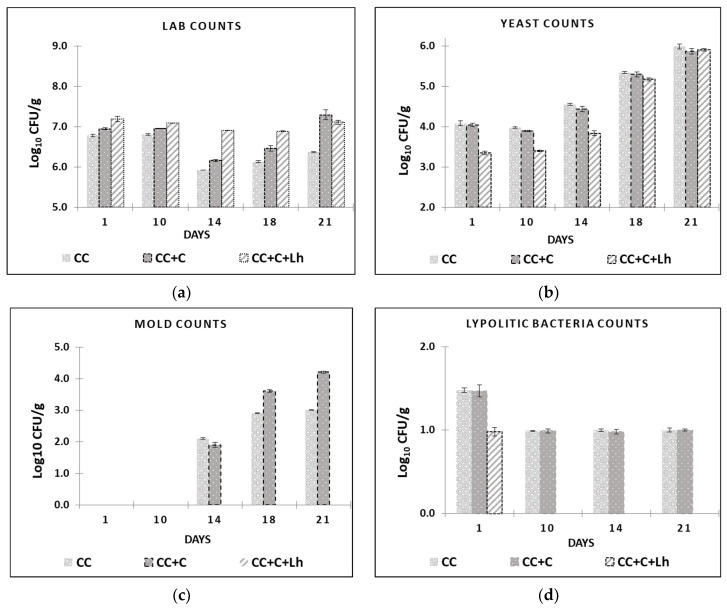
Lactic acid bacteria (LAB) (**a**), yeast (**b**), and mould (**c**) counts and lipolytic bacteria (**d**), are presented for control acid-curd cheese (CC), coated acid-curd cheese (CC + C), and coated acid-curd cheese with *L. helveticus* (CC + C + Lh) during 23 days of storage at 4–6 °C (mean values ± SD).

Low pH (4.3–4.8), distinctive wedge-like shape with semi-soft texture, and whitish colour are the main qualities of acid-curd cheese. The variation in pH as storage time elapsed is depicted in Figure 2c. At day 1, pH in all samples ranged from 4.24 (CC) to 4.27 (CC + C + Lh) and 4.29 (CC + C), indicating a small but significant impact of acid whey base coating on the curd cheese pH (*p* < 0.05). In a course of naturally occurring proteolysis [1,45], pH values increased and then decreased in all cheese samples. This process was faster in case of coated samples reaching the pH peak on day 14 compared with control ones reaching it on day 18. Between coated samples, the ripening process was more intense in the sample with lactobacilli incorporated into the coating (4.56 ± 0.05 for CC + C and 4.71 ± 0.06 for CC + C + Lh, *p* < 0.05). We can speculate that the coating presence contributed to cheese proteolysis by preserving the moisture in cheese (Figure 2b) and thus favouring the growth or survival of LAB (Figure 3a) that resulted in significantly lower pH (Figure 2c) and higher acidity values (Figure 2d; (*p* < 0.05)). Furthermore, the addition of *L. helveticus* strain to the coating significantly sped up the above-mentioned process (*p* < 0.01), showing that the immobilisation in coating may be an effective way for microorganism survival [26].

The concentration of lactic acid (g/100 g) is presented in Figure 2d. At day 1, the concentration of lactic acid in fresh control curd cheeses (CC) was higher (2.15± 0.11; *p* < 0.05) compared with coated CC + C (1.52 ± 0.01) and CC + C + Lh (1.52 ± 0.02) samples; the samples switched places from day 14 with coated samples demonstrating higher (*p* < 0.05) lactic acid content compared with the control one, indicating growth of lactic acid bacteria in this cheese. Lactic acid produced by LAB consequently helps to draw out moisture from the cheese mass [46], and therefore, we observed more intense moisture (Figure 2b) and weight loss (Figure 2a of uncoated cheese (CC)) from day 10 compared with coated ones CC + C and CC + C + Lh (*p* < 0.05). Moreover, the moisture loss in the uncoated sample led to the harder texture (Figure 2e; *p* < 0.01) and discoloration (Figure 2f; *p* < 0.001). Cheese coated with plain coating (CC + C) lost the least weight, had the softest texture, and demonstrated the lowest change in colour compared with the other samples (*p* < 0.05). Addition of *L. helveticus* to the coating not only significantly reduced moisture-retaining properties of the coating but also contributed to cheese weight loss and colour change. It was reported by Ma, Jiang, Ahmed, Quin, and Liu (2018) [44] that the addition of LAB can change the spatial structure of the molecules, destroy intermolecular interactions, and enlarge the intermolecular space, which could decrease coating’s barrier properties. Moisture loss in curd cheese visually results in its colour change from white to yellow due to surface drying. Colour is an important sensory attribute of food products, and thus, consumers avoid cheese that is discoloured [47]. Instrumental colour determination revealed that, at day 1, there were no significant differences among samples. During the storage, however, uncoated control samples had significantly (*p* < 0.05) higher values of b* coordinates (data not shown) than coated ones, which means that uncoated samples were more yellow than coated counterparts. Overall colour change (∆E, calculated by comparing L*a*b* values of day 1 with values of days 10, 14, 18, and 21) of cheeses during storage is shown in Figure 2f. Uncoated samples showed the highest changes in colour during all storage. Between coated samples, CC + C had significantly lower (*p* < 0.05) ∆E values than the bacteria-supplemented one (CC + C + Lh). With regard to our previous study on liquid whey protein (LWPC) coated acid-curd cheese, our recent findings allow us to conclude that liquid sweet and acid whey protein-based coatings have similar potential to improve cheese quality by preserving moisture, and naturally cheese colour and appearance [43].

#### 3.2.2. Microbiological Profile

Mesophilic LAB along with lesser counts of potential spoilage microorganisms such as enterococci, enterobacteria, and yeast and mould can be present in fresh artisanal acid-curd cheese [48]. Many manual manufacturing operations and absence of modern plastic packaging by artisanal cheese processors negatively affect cheese hygienic quality and end up with short shelf life (8–10 days). In our study, 1–1.5 log_10_ CFU/g of coliforms and enterobacteria were detected in all cheeses on day 1, with no statistical difference among samples (*p* > 0.05, data not shown). During the rest of the storage period, these indicator microorganisms were no longer detectable (<1 log_10_ CFU/g) in any of the cheese samples.

The results of mesophilic LAB, lipolytic bacteria, yeast, and mould are shown in Figure 3. Initial mesophilic LAB counts were 7.5 log_10_ CFU/g for control cheese. These counts were similar to those reported for similar cheese types [43,48]. Total LAB counts dropped down for approx. 1 log_10_ unit in control (CC) and plain coated (CC + C) cheese samples on day 14 (Figure 3a). From there, these samples underwent a slight but significant increase (*p* ≤ 0.05) in LAB counts, registering close to 6.4 log_10_ CFU/g for control (CC) and 7,3 log_10_ CFU/g for plain coated CC + C at the end of the storage. No effect of storage on LAB counts was observed in *L. helveticus* supplemented sample (CC + C + Lh), but in coated cheeses, LAB counts increased after day 14 and were significantly higher than the control cheese (*p* ≤ 0.05) at the end of storage.

Lipolytic microorganisms (aerobic and anaerobic bacteria of the genera *Pseudomonas* and *Clostridium*) and mould fungi are the most common contaminants of dairy products responsible for visible or non-visible defects, such as off-odour and off-flavour [49]. Lipolytic microorganisms cause damage to food products; thus, according to the instruction of microbiological control [50], it is recommended for dairy factories to not exceed 2 log_10_ units CFU/g limit of such psychrotrophic spoilage microflora. In our study, significant inhibition of spoilage microorganisms was achieved by protective strain incorporated in the coating (Figure 3b–d). Our coating with *L. helveticus* (CC + C + Lh) was able to suppress the growth of yeast for up to 1.0 log_10_ units for 14 days of storage (*p* ≤ 0.05) and prevent mould growth for 21 days compared with other samples where the mould appeared on cheese surface as early as day 14 (Figure 2c). It also suppressed lipolytic bacteria growth on day 1; these microorganisms were no longer detectable (<1 log_10_ CFU/g) in CC + C + Lh samples during the rest of the storage period compared with control and plain coated cheeses samples (1.5–1 log_10_ CFU/g; Figure 3d). This is in agreement with Guimarães et al. (2020) [51], where whey protein coatings containing *L. buchneri* cells prevented microbial spoilage in cheese for at least 30 days.

#### 3.2.3. Sensory Profile

**Figure 4:**
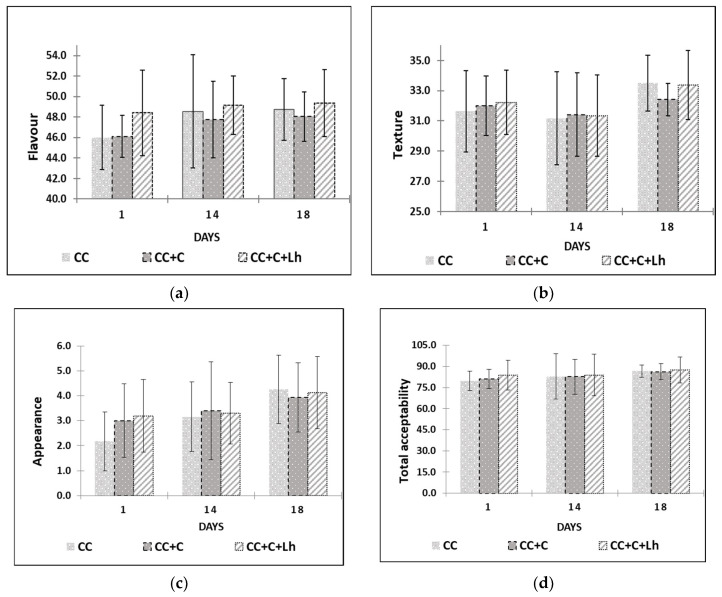
Flavour (**a**), body and texture (**b**), appearance (**c**), and overall acceptability (**d**) are presented for control curd cheese (CC), coated curd cheese (CC + C), and coated cheese with *L. helveticus* (CC + C + Lh) during 18 days of sensory evaluation (mean values ± SD).

Edible films and coatings can contain flavouring substances, improving the sensory properties of foods [16]; thus, through sensory trials (Figure 4), we intended to assess the influence of our edible coatings on flavour, body and texture, appearance, and overall acceptability of acid-curd cheese. The results of the factorial analysis revealed no single treatment factor impact on any of sensory parameters of curd cheese. The storage day factor and storage and treatment interaction, though, had a significant impact on the perception of cheese flavour (the interaction factor; *p* ≤ 0.001), on overall acceptability, and on cheese texture (storage factor; *p* ≤ 0.01).

The scores of overall acceptability, flavour, and texture increased throughout the storage of cheese (*p* ≤ 0.05). Cheese samples coated with *L. helveticus* (CC + C + Lh) received the highest scores for flavour throughout the storage (*p* ≤ 0.001) compared with other samples. Plain coating based on sweet whey protein concentrate when used on the same type of cheese in our previous study [43] did not indicate any significant effect of the coating on the flavour of cheese. Fresh acid-curd cheese is known for slight dull and indistinctive flavour. Addition of sweet and sour tasting coating with lactobacilli incorporated positively affected cheese flavour and was described by the panellists as “more tasteful”. During the storage, the growth of spoilage microflora in control (CC) and in cheese with plain coating (CC + C) resulted in various off-odours and off-flavours lowering scores of the flavour parameter. Sensory defects in fresh curd cheese are very often caused by microbial contamination [49]; thus, bioprotective LAB cultures are employed to prevent the spoilage and preserve the flavour of the product.

With regard to appearance, coated cheese samples were perceived as better (*p* ≤ 0.05) than those of uncoated samples in the beginning of storage due to “fresher look” noted by the panellists. There were no significant differences among samples at the end of testing on day 18 in neither appearance nor total acceptability.

## 4. Conclusions

The presence of antimicrobial edible coating on fresh acid-curd cheeses was evaluated in order to represent small-scale East European acid-curd cheese producers that sell cheese unpacked or only wrapped in paper. Applied coating significantly improved appearance and slowed down colour changes by preserving moisture in cheese. The coating with incorporated indigenous *L. helveticus* decreased the growth of spoilage microorganisms during prolonged cheese storage, thus significantly improving the flavour of cheese.

In general, the incorporation of LAWPC as an acid-curd cheese by-product back into dairy production as a base of edible coating is quite beneficial for both small and big scale manufacturers. LAWPC-based edible coating could be prepared immediately after acid-curd cheese production reusing leftover whey and other agro-industrial waste ingredients obtained with minimal investments. This type of active edible coating with antimicrobial effect could be an excellent addition to both package-free and vacuum-packaged cheeses produced by manufacturers aiming for sustainability, enhanced quality, and extended shelf life of the final product.

## Figures and Tables

**Table 1:** Chemical compositions of curd cheese treatments during cold storage.

	D1			D23		
Content	CC	CC + C	CC + C + Lh	CC	CC + C	CC + C + Lh
Dry matter, %	33.74 ± 0.83 *	34.29 ± 0.82 *	34.69 ± 1.49 *	57.21 ± 0.78 *	43.63 ± 1.41 B *	45.52 ± 0.6 C *
Fat,%	0.56 ± 0.03 A *	0.65 ± 0.01	0.70 ± 0.00 B *	1.26 ± 0.03 A *	1.35 ± 0.01 *	1.52 ± 0.01 B *
Protein, %	30.62 ± 0.03 *	30.12 ± 0.01 *	30.28 ± 0.01 *	46.62 ± 0.03 *	40.72 ± 0.01 B *	40.16 ± 0.02 B *
Lactose, %	2.49 ± 0.04 A *	3.03 ± 0.01 *	3.22 ± 0.00 B *	1.39 ± 0.04 A *	1.42 ± 0.01 A *	1.22 ± 0.02 B *

Means between storage days within same cheese treatment marked with asterisk (*) are significantly different (*p* ≤ 0.05). Means between cheese treatments within same storage day marked with different capital letters (A–C) are significantly different (*p* ≤ 0.05). Control acid-curd cheese (CC), coated acid-curd cheese (CC + C), coated acid-curd cheese with *L. helveticus* (CC + C + Lh).

## Data Availability

Not applicable.
